# Antiinflammatory Activity of Cinnamon (Cinnamomum zeylanicum) Bark Essential Oil in a Human Skin Disease Model

**DOI:** 10.1002/ptr.5822

**Published:** 2017-04-26

**Authors:** Xuesheng Han, Tory L. Parker

**Affiliations:** ^1^ dōTERRA International, LLC 389 S. 1300 W Pleasant Grove UT 84062 USA

**Keywords:** cinnamon bark essential oil, cell proliferation, inflammation, VCAM‐1, MIG, genome‐wide gene expression

## Abstract

The effect of cinnamon (Cinnamomum zeylanicum) bark essential oil (CBEO) on human skin cells has not been elucidated. Therefore, we investigated the activity of a commercially available CBEO in a validated human dermal fibroblast system, a model of chronic inflammation and fibrosis. We first evaluated the impact of CBEO on 17 protein biomarkers that play critical roles in inflammation and tissue remodeling. The impact of CBEO on genome‐wide gene expression was also evaluated. CBEO showed strong anti‐proliferative effects on skin cells and significantly inhibited the production of several inflammatory biomarkers, including vascular cell adhesion molecule‐1, intercellular cell adhesion molecule‐1, monocyte chemoattractant protein‐1, interferon gamma‐induced protein 10, interferon‐inducible T‐cell alpha chemoattractant, and monokine induced by gamma interferon. In addition, CBEO significantly inhibited the production of several tissue remodeling molecules, including epidermal growth factor receptor, matrix metalloproteinase‐1, and plasminogen activator inhibitor‐1. Macrophage colony‐stimulating factor, which is an immunomodulatory protein molecule, was also significantly inhibited by CBEO. Furthermore, CBEO significantly modulated global gene expression and altered signaling pathways, many of which are important in inflammation, tissue remodeling, and cancer biology. The study shows that CBEO is a promising antiinflammatory agent; however, further research is required to clarify its clinical efficacy. © 2017 The Authors. Phytotherapy Research published by John Wiley & Sons Ltd.

## Introduction

Cinnamon (Cinnamomum zeylanicum) bark essential oil (CBEO) has been used for thousands of years in Ayurvedic medicine to soothe aching joints and numb pain. It is still used for similar purposes in India, presumably because of its antiinflammatory property. CBEO typically contains a very high amount of cinnamaldehyde and a small amount of eugenol, among many other aromatic compounds. CBEO and cinnamaldehyde have been studied for their antibacterial (Bardají *et al*., 2016), antifungal (Ranasinghe *et al*., 2002), anti‐diabetic (Anderson *et al*., 2013; Sartorius *et al*., 2014), antiinflammatory (Mendes *et al*., 2016; Chen *et al*., 2016), and anticancer (Yang *et al.,* 2015) activities, among others.

Furthermore, CBEO has gained popularity for use in skin care products; however, research on its effects on human skin is largely scarce. A recent study (Uchi *et al*., 2017) conducted on human keratinocytes demonstrated the antioxidant effect of cinnamaldehyde, as well as its potential for treating skin disorders. Therefore, we aimed to investigate the biological activity of a commercially available CBEO in a human dermal fibroblast system, which was designed to mimic chronic inflammation and fibrosis. We first evaluated the impact of CBEO on 17 protein molecules that are relevant to the processes of inflammation, immune response, and tissue remodeling. We also studied the effects of CBEO on genome‐wide gene expression. The study provides important evidence of the biological activity of CBEO in a human skin disease model.

## Materials and Methods

All experiments were conducted in a Biologically Multiplexed Activity Profiling system HDF3CGF, a cell culture of human dermal fibroblasts that is designed to model chronic inflammation and fibrosis in a robust and reproducible way. The system consists of three components: a cell type, stimuli to create the disease environment, and set of biomarker (protein) readouts to examine how treatments affect that disease environment (Berg *et al.,* 2010). The methodologies used in this study were essentially the same as those previously described (Han and Parker, 2017a, 2017b; Han *et al.,* 2017).

### Reagents

Cinnamon bark essential oil (dōTERRA International LLC, Pleasant Grove, UT, USA) was diluted in DMSO to 8X the specified concentrations [final DMSO concentration in culture media was no more than 0.1% (*v*/*v*)]; 25 μL of each 8X solution was added to the cell culture to a final volume of 200 μL. DMSO [0.1% (*v*/*v*)] served as the vehicle control. Gas chromatography–mass spectrometry analysis of CBEO indicated that its major chemical constitutes (i.e., >5%) were *trans*‐cinnamaldehyde (59%) and cinnamyl acetate (15%). The original gas chromatography–mass spectrometry chromatogram is shown in Fig. S1, and a list of all chemical constitutes found in CBEO is provided in Table S1.

### Cell cultures

Primary human neonatal fibroblasts were obtained as previously described (Bergamini *et al*., 2012) and were plated under low serum conditions (0.125% fetal bovine serum) for 24 h. Then, the cell culture was stimulated with a mixture of interleukin‐1β, tumor necrosis factor‐α, interferon‐γ, basic fibroblast growth factor, epidermal growth factor, and platelet‐derived growth factor, for another 24 h. The cell culture and stimulation conditions for the HDF3CGF assays have been described in detail elsewhere and were performed in a 96‐well format (R Development Core Team, 2011; Bergamini *et al.,* 2012).

### Protein‐based readouts

An enzyme‐linked immunosorbent assay was used to measure the biomarker levels of cell‐associated and cell membrane targets. Soluble factors from supernatants were quantified using homogeneous time‐resolved fluorescence detection, bead‐based multiplex immunoassay, or captured enzyme‐linked immunosorbent assay. Overt adverse effects of the test agents on cell proliferation and viability (cytotoxicity) were measured using SRB assay. For proliferation assays, cells were cultured and then assayed after 72 h, which was optimized for the HDF3CGF system. Detailed information has been described elsewhere (Bergamini *et al.,* 2012). Measurements were performed in triplicate wells, and a glossary of the biomarkers used in this study is provided in Table S2.

Quantitative biomarker data are presented as the mean log_10_ relative expression level (compared with the respective mean vehicle control value) ± standard deviation of triplicate measurements. Differences in biomarker levels between CBEO‐treated and vehicle‐treated cultures were tested for significance with the unpaired Student's *t*‐test. A *p*‐value <0.01, outside of the significance envelope, with an effect size of at least 20% (more than 0.1 log_10_ ratio units), was considered statistically significant.

### RNA isolation

Total RNA was isolated from cell lysates using the Zymo *Quick‐RNA* MiniPrep kit (Zymo Research Corporation, Irvine, CA, USA), according to manufacturer's instructions. RNA concentration was determined using NanoDrop ND‐2000 (Thermo Fisher Scientific, Waltham, MA, USA). RNA quality was assessed with a Bioanalyzer 2100 (Agilent Technologies, Santa Clara, CA, USA) and an Agilent RNA 6000 Nano Kit. All samples had an A260/A280 ratio between 1.9 and 2.1, and an RNA integrity number score greater than 8.0.

### Microarray analysis for genome‐wide gene expression

A 0.0012% (*v*/*v*) concentration of CBEO was tested for its effect on expression of 21,224 genes in the HDF3CGF system after 24 h treatment. Samples for microarray analysis were processed by Asuragen, Inc. (Austin, TX, USA), according to the company's standard operating procedures. Biotin‐labeled cRNA was prepared from 200 ng of total RNA with an Illumina TotalPrep RNA Amplification kit (Thermo Fisher Scientific) and one round of amplification. The cRNA yields were quantified via UV spectroscopy, and the distribution of transcript sizes was assessed using the Agilent Bioanalyzer 2100. Labeled cRNA (750 ng) was used to probe Illumina Human HT‐12 v4 Expression BeadChips (Illumina, Inc., San Diego, CA, USA). Hybridizing, washing, staining with streptavidin‐conjugated Cyanine‐3, and scanning of the Illumina arrays was performed according to the manufacturer's instructions. Illumina BeadScan software was used to produce the data files for each array; raw data were extracted using Illumina BeadStudio software.

Raw data were uploaded into R (R Development Core Team, 2011) and analyzed for quality‐control metrics using the *beadarray* package (Dunning *et al.,* 2007). Data were normalized using quantile normalization (Bolstad *et al.,* 2003), then re‐annotated and filtered to remove probes that were non‐specific or mapped to intronic or intragenic regions (Barbosa‐Morais *et al.,* 2010). The remaining probe sets comprised the data set for the remainder of the analysis. Fold‐change expression for each value was calculated as the log_2_ ratio of CBEO to vehicle control. These fold‐change values were uploaded to Ingenuity Pathway Analysis (IPA, Qiagen, Redwood City, CA, www.qiagen.com/ingenuity) to generate the network and pathway analyses.

## Results and Discussion

### Bioactivity profile of CBEO in pre‐inflamed human dermal fibroblasts

**Figure 1:**
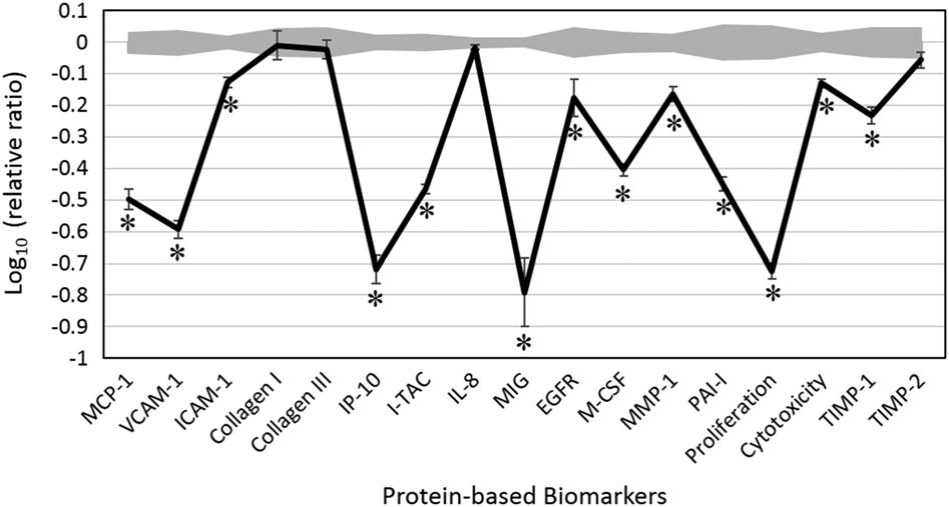
The bioactivity profile of cinnamon bark essential oil (0.0012% *v*/*v*) in the human dermal fibroblast system HDF3CGF. The *x*‐axis denotes protein‐based biomarker readouts. The *y*‐axis denotes the relative expression levels of biomarkers when compared with the values for the vehicle control in log form. Vehicle control values are marked in gray at a 95% confidence level. * indicates a biomarker designated as having ‘key activity’, which is when a biomarker value is significantly different (*p* < 0.01) from the respective value for the vehicle control at a studied concentration, with an effect size of at least 20% (more than 0.1 log ratio units). MCP‐1, monocyte chemoattractant protein; VCAM‐1, vascular cell adhesion molecule 1; ICAM‐1, intracellular cell adhesion molecule 1; IP‐10, interferon gamma‐induced protein 10; I‐TAC, interferon‐inducible T‐cell alpha chemoattractant; IL‐8, interleukin‐8; MIG, the monokine induced by gamma interferon; EGFR, epidermal growth factor receptor; M‐CSF, macrophage colony‐stimulating factor; MMP‐1, matrix metalloproteinase 1; PAI‐1, plasminogen activator inhibitor 1; TIMP, tissue inhibitor of metalloproteinase.

We analyzed CBEO's biological activity in a dermal fibroblast cell system, HDF3CGF, which mimics the disease microenvironment of inflamed human skin cells with already stimulated immune responses. Four different concentrations (0.011, 0.0037, 0.0012, and 0.00041% *v*/*v*, using DMSO as solvent) of CBEO were initially studied for their effects on cell viability. The two high concentrations were overtly cytotoxic; thus, only the 0.0012% *v*/*v* preparation, which contained the highest concentration of CBEO that was non‐cytotoxic, was used in further analyses. Key activities of biomarkers were indicated when biomarker values for test samples were significantly different (*p* < 0.01) from the respective values for the vehicle controls, with an effect size of at least 20% (more than 0.1 log ratio units) (Fig. 1).

Cinnamon bark essential oil inhibited all the 17 biomarkers that were studied. It showed a significant anti‐proliferative activity against the dermal fibroblast cells. CBEO also significantly inhibited the production of inflammatory cytokines such as monocyte chemoattractant protein‐1, interferon gamma‐induced protein 10, interferon‐inducible T‐cell alpha chemoattractant, and monokine induced by gamma interferon (MIG). Furthermore, CBEO significantly inhibited the production of vascular cell adhesion molecule‐1 (VCAM‐1) and intercellular cell adhesion molecule‐1. The levels of several tissue remodeling molecules, including epidermal growth factor receptor (EGFR), matrix metalloproteinase 1, and plasminogen activator inhibitor‐1, were significantly decreased by CBEO. CBEO also significantly reduced the levels of macrophage colony‐stimulating factor, which is an immunomodulatory protein. The strong inhibitory effect of CBEO on the increased production of these biomarkers indicates that it may have an antiinflammatory property and, therefore, promote wound healing.

Previous studies have indicated that CBEO and its major active component cinnamaldehyde may have promising antiinflammatory properties (Tung *et al.,* 2008; Wang *et al.,* 2015). Chen *et al.* (2016) demonstrated using an animal model that trans‐cinnamaldehyde suppresses lipopolysaccharide‐induced inflammation (Chen *et al.,* 2016). Another study has similarly reported on the antiinflammatory activity of cinnamaldehyde (Mendes *et al*., 2016). The observed strong antiinflammatory effect of CBEO in the human skin disease model suggests that CBEO may be used for treating inflammatory skin conditions.

In addition, the inhibitory effect of CBEO on the production of tissue remodeling biomarkers in the highly inflamed skin model suggests that CBEO and cinnamaldehyde may be beneficial for wound healing. This might be, at least, partially attributed to their antiinflammatory and antimicrobial properties (Ghosh *et al.,* 2013; Wang *et al.,* 2015).

### Effects of CBEO on genome‐wide gene expression

To further explore the biological activities of CBEO on human skin cells, we studied the effect of the highest concentration of CBEO that was not cytotoxic to the cells (0.0012% *v*/*v*) on the RNA expression of 21,224 genes in the HDF3CGF system. The results showed a significant, strong, and diverse effect of CBEO on the regulation of the genes. Of the 200 genes that were highly regulated by CBEO [log_2_ (expression fold‐change ratio relative to vehicle control) ≥ |1.5|], 148 genes were significantly downregulated, whereas 52 genes were significantly downregulated (Table S3). A cross comparison of protein and gene expression data showed that CBEO significantly inhibited the protein and gene expression levels of MIG and VCAM‐1.

**Figure 2:**
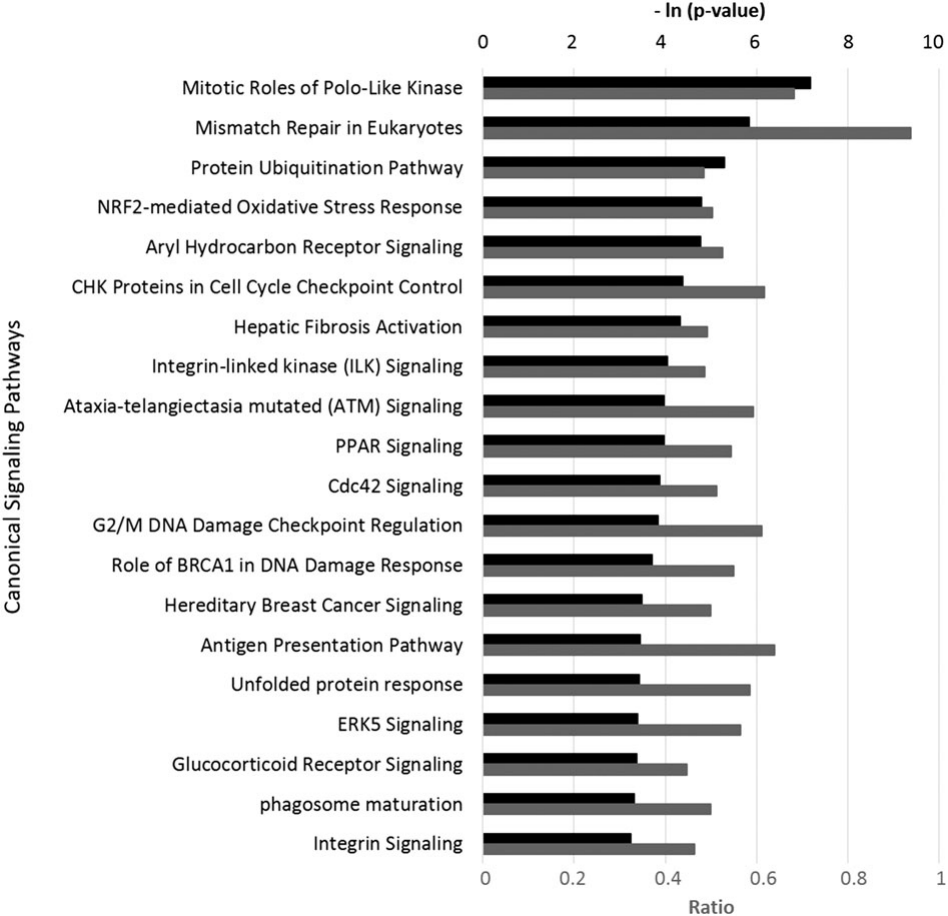
Top 20 canonical pathways matching the bioactivity profile of cinnamon bark essential oil (0.0012% *v*/*v*) in gene expression in the HDF3CGF system, as obtained from the IPA. The *p*‐values were calculated using right‐tailed Fisher's exact test. Each *p*‐value measures how likely an observed association between a specific pathway and the dataset would be if it were only due to a random chance. The smaller the *p*‐value [bigger – ln (p‐value), indicated by the black bars] for a pathway is, the more significantly it matches with the bioactivity of CBEO. A ratio, indicated by the gray bar, was calculated by dividing the number of genes from the CBEO dataset that participated in a canonical pathway by the total number of genes in that pathway. NRF2, nuclear factor E2‐related factor 2; CHK, checkpoint kinase; PPAR, peroxisome proliferator‐activated receptor; Cdc 42, cell division control protein 42 homolog; BRCA1, breast cancer type 1 susceptibility protein; ERK5, extracellular signal‐regulated kinase 5.

The IPA showed that the bioactivity of CBEO significantly overlapped with many canonical signaling pathways from literature‐validated databases (Fig. 2). Some of these top signaling pathways (Table S4–S7), such as the hepatic fibrosis activation pathway and the antigen presentation pathway, are critically involved in inflammation and tissue remodeling. Interestingly, many pathways that play critical roles in cell cycle regulation, cancer biology, and DNA damage response were also on the list. The overall inhibitory effect of CBEO on the genes studied and the signaling pathways is not only consistent with its antiinflammatory and immune modulating potential; however, it also suggests that CBEO may play a role in modulating cell cycle regulation and cancer signaling.

Cinnamon essential oil has been reported to show a significant anticancer activity against head and neck squamous cell carcinoma via the suppression of EGFR tyrosine kinase (Yang *et al.,* 2015). The EGFR tyrosine kinase signaling pathway is important for the growth, survival, proliferation, and differentiation of cells (Oda *et al.,* 2005). Cinnamaldehyde has also been reported to be a potential anticancer drug (Hong *et al.,* 2016), primarily due to its anti‐mutagenic, anti‐tumorigenic, anti‐proliferative, and pro‐apoptotic properties in cancer cell lines. The findings of our study are largely consistent with the anticancer properties of CBEO.

The current study has limitations. The *in vitro* study results cannot be directly extrapolated to more complex human skin systems. Moreover, the impact of CBEO on human global gene expression was measured over a short period. Therefore, how CBEO affects long‐term gene expression remains elusive. Nevertheless, the data from the current study provide important evidence of the biological activities of CBEO in human skin cells and suggest that CBEO is a promising antiinflammatory agent.

## Conclusions

To our knowledge, this is the first study to investigate the biological activity of CBEO in a human skin disease model. We found that CBEO significantly inhibited the production of several protein biomarkers that are involved in inflammation and tissue remodeling. Moreover, CBEO showed significant anti‐proliferative activity against the skin cells used in the study. Genome‐wide gene expression analysis demonstrated that CBEO significantly modulated global gene expression and some signaling pathways. It was noted that many of the genes and signaling pathways affected by CBEO play critical roles in inflammation, immune response, cancer biology, and DNA damage response. The overall inhibitory effect of CBEO suggests its potential in regulating the abovementioned biological processes. However, further research is needed to evaluate the mechanism of action of CBEO, as well as its clinical efficacy and safety.

## Conflict of Interest

X.H. and T.P. are employees of dōTERRA, the manufacturer of the CBEO used in the study.

## Supporting information


**Fig. S1**. The chromatogram of cinnamon bark essential oil (CBEO) analyzed by gas chromatography–mass spectrometry (GC–MS).
**Table S1**. Chemical composition of cinnamon bark essential oil (CBEO) analyzed by gas chromatography–mass spectrometry (GC–MS).
**Table S2**. Glossary of biomarkers of system HDF3CGF used in the study.
**Table S3**. The 200 genes most‐impacted by cinnamon bark essential oil (CBEO).
**Table S4**. Top 20 genes regulated by CBEO in the canonical Mitotic Roles of Polo‐Like Kinase pathway. Fold change over vehicle was shown in log2 ratio form.
**Table S5**. Top 15 genes regulated by CBEO in the canonical Mismatch Repair in Eukaryotes pathway. Fold change over vehicle was shown in log2 ratio form.
**Table S6**. Top 20 genes regulated by CBEO in the canonical Protein Ubiquitination pathway. Fold change over vehicle was shown in log2 ratio form.
**Table S7**. Top 20 genes regulated by CBEO in the canonical NRF2‐mediated Oxidative Stress Response pathway. Fold change over vehicle was shown in log2 ratio form.
