# Supplementary material for: Antiinflammatory Activity of Cinnamon (Cinnamomum zeylanicum) Bark Essential Oil in a Human Skin Disease Model
Source: Phytother Res. 2017 Apr 26;31(7):1034–8. doi: 10.1002/ptr.5822 (PMC5518441; doi:10.1002/ptr.5822)

**Supplementary Material**

Figure S1. The chromatogram of cinnamon bark essential oil (CBEO) analyzed by gas chromatography–mass spectrometry (GC-MS)

Table S1. Chemical composition of cinnamon bark essential oil (CBEO) analyzed by gas chromatography–mass spectrometry (GC-MS)

| **Retention Time** | **Compound Name** | **Area Sum %** |
| --- | --- | --- |
| 6.68 | Styrene | 0.21 |
| 8.341 | Thujene <alpha-> | 0.17 |
| 8.618 | Pinene <alpha> | 1.00 |
| 9.398 | Camphene | 0.47 |
| 10.085 | Benzaldehyde | 0.22 |
| 10.751 | Sabinene | 0.04 |
| 10.905 | Pinene <beta> | 0.32 |
| 11.931 | Myrcene | 0.08 |
| 12.69 | Phellandrene <alpha> | 1.12 |
| 12.803 | 3-Carene | 0.09 |
| 13.357 | Terpinene -alpha | 0.94 |
| 13.828 | p-Cymene | 2.19 |
| 14.126 | .beta.-Phellandrene | 3.78 |
| 14.218 | Eucalyptol | 0.36 |
| 14.844 | Ocimene <Z,beta> | 0.06 |
| 16.013 | Terpinene <gamma> | 0.14 |
| 16.3 | Acetophenone | 0.46 |
| 17.726 | Terpinolene | 0.14 |
| 18.998 | Linalool | 2.08 |
| 20.31 | Menth-2-en-1-ol <cis,p> | 0.07 |
| 21.572 | Camphor | 0.40 |
| 22.823 | Hydrocinnamaldehyde | 0.43 |
| 23.367 | Borneol | 0.20 |
| 24.054 | Terpinen-4-ol | 0.66 |
| 24.741 | p-Cymen-8-ol | 0.06 |
| 25.295 | Terpineol <alpha> | 0.73 |
| 25.828 | Carveol isomer | 0.08 |
| 27.049 | Cinnamaldehyde Z | 0.32 |
| 28.28 | Hydrocinnamyl alcohol | 0.10 |
| 32.075 | Cinnamaldehyde, (E)- | 59.42 |
| 35.254 | Cinnamyl alcohol <E> | 0.46 |
| 39.829 | Eugenol | 2.64 |
| 41.798 | Copaene <alpha> | 0.68 |
| 46.003 | Caryophyllene <beta> | 2.49 |
| 49.757 | Humulene <alpha> | 0.44 |
| 50.106 | Cinnamyl acetate <(E)-> | 15.04 |
| 57.285 | Cadinene <delta> | 0.12 |
| 58.239 | Methoxycinnamaldehyde-o | 0.32 |
| 62.362 | Palustrol | 0.23 |
| 62.988 | Caryophyllene Oxide | 0.49 |
| 73.193 | Aristolene isomer | 0.09 |
| 78.157 | Benzyl Benzoate | 0.64 |

Table S2. Glossary of biomarkers of system HDF3CGF used in the study

| **Readout** | **Description** |
| --- | --- |
| **CCL2/MCP-1** | MCP-1 system is a chemokine that mediates recruitment of monocytes and T cells into sites of inflammation. MCP-1 is categorized as an inflammation-related activity in the HDF3CGF system modeling Th1 inflammation involved in wound healing and matrix remodeling. |
| **CD106/VCAM-1** | VCAM-1 is a cell adhesion molecule that mediates adhesion of monocytes and T cells to endothelial cells. VCAM-1 is categorized as an inflammation-related activity. |
| **CD54/ICAM-1** | ICAM-1 is a cell adhesion molecule that mediates leukocyte-endothelial cell adhesion and leukocyte recruitment. ICAM-1 is categorized as an inflammation-related activity. |
| **Collagen I** | Collagen I is involved in tissue remodeling and fibrosis, and is the most common fibrillar collagen that is found in skin, bone, tendons and other connective tissues. Collagen I is categorized as a tissue remodeling-related activity. |
| **Collagen III** | Collagen III is an extracellular matrix protein and fibrillar collagen found in extensible connective tissues (skin, lung and vascular system) and is involved in cell adhesion, cell migration, tissue remodeling. Collagen III is categorized as a tissue remodeling-related activity. |
| **CXCL10/IP-10** | IP-10 is a chemokine that mediates T cell, monocyte and dendritic cell chemotaxis. IP-10 is categorized as an inflammation-related activity. |
| **CXCL11/I-TAC** | I-TAC is a chemokine that mediates T cell and monocyte chemotaxis. I-TAC is categorized as an inflammation-related activity. |
| **CXCL8/IL-8** | IL-8 is a chemokine that mediates neutrophil recruitment into acute inflammatory sites. IL-8 is categorized as an inflammation-related activity. |
| **CXCL9/MIG** | MIG is a chemokine that mediates T cell recruitment. MIG is categorized as an inflammation-related activity. |
| **EGFR** | EGFR is a cell surface receptor for epidermal growth factor involved in cell proliferation during development as well as tumor growth. EGFR is involved in Epithelial cell proliferation, epithelial cell differentiation keratinocyte proliferation, tissue remodeling. EGFR is categorized as a tissue remodeling-related activity. |
| **M-CSF** | M-CSF is a secreted and cell surface cytokine that mediates macrophage differentiation. M-CSF is categorized as an immune modulation-related activity. |
| **MMP-1** | MMP-1 is an interstitial collagenase that degrades collagens I, II and III and is involved in the process of tissue remodeling. MMP-1 is categorized as a tissue remodeling-related activity. |
| **PAI-I** | PAI-I is a serine proteinase inhibitor and inhibitor of tissue plasminogen activator (tPA) and urokinase (uPA) and is involved in tissue remodeling and fibrinolysis. PAI-I is categorized as a tissue remodeling-related activity. |
| **Proliferation_72hr** | Proliferation_72hr in the HDF3CGF system is a measure of dermal fibroblast proliferation which is important to the process of wound healing and fibrosis. |
| **SRB** | SRB is a measure of the total protein content of dermal fibroblasts. Cell viability of adherent cells is measured by Sulforhodamine B (SRB) staining, a method that determines cell density by measuring total protein content of test wells. |
| **TIMP-1** | TIMP-1 is a tissue inhibitor of matrix metalloprotease-7 (MMP-7) and other MMPs, and is involved in tissue remodeling, angiogenesis and fibrosis. TIMP-1 is categorized as a tissue remodeling-related activity. |
| **TIMP-2** | TIMP-2 is a tissue inhibitor of matrix metalloproteases and is involved in tissue remodeling, angiogenesis and fibrosis. TIMP-2 is categorized as a tissue remodeling-related activity. |

Table S3. The 200 genes most-impacted by cinnamon bark essential oil (CBEO)

| **Illumina Gene ID** | **Fold Change in Log_2_ form** | **Definition** |
| --- | --- | --- |
| SLC7A11 | 34.48 | Homo sapiens solute carrier family 7, (cationic amino acid transporter, y+ system) member 11 (SLC7A11), mRNA. |
| AKR1C4 | 32.87 | Homo sapiens aldo-keto reductase family 1, member C4 (chlordecone reductase; 3-alpha hydroxysteroid dehydrogenase, type I; dihydrodiol dehydrogenase 4) (AKR1C4), mRNA. |
| HMOX1 | 25.70 | Homo sapiens heme oxygenase (decycling) 1 (HMOX1), mRNA. |
| HSPA6 | 21.81 | Homo sapiens heat shock 70kDa protein 6 (HSP70B') (HSPA6), mRNA. |
| SLCO2B1 | 20.20 | Homo sapiens solute carrier organic anion transporter family, member 2B1 (SLCO2B1), mRNA. |
| TREML3 | 15.96 | PREDICTED: Homo sapiens triggering receptor expressed on myeloid cells-like 3 (TREML3), mRNA. |
| AKR1C2 | 15.44 | Homo sapiens aldo-keto reductase family 1, member C2 (dihydrodiol dehydrogenase 2; bile acid binding protein; 3-alpha hydroxysteroid dehydrogenase, type III) (AKR1C2), transcript variant 1, mRNA. XM_943424 XM_943425 XM_943427 |
| GLDN | 12.31 | Homo sapiens gliomedin (GLDN), mRNA. |
| MIR221 | 11.61 | Homo sapiens microRNA 221 (MIR221), microRNA. |
| SRXN1 | 11.32 | Homo sapiens sulfiredoxin 1 homolog (S. cerevisiae) (SRXN1), mRNA. |
| F2RL2 | 10.96 | Homo sapiens coagulation factor II (thrombin) receptor-like 2 (F2RL2), mRNA. |
| GDF15 | 10.90 | Homo sapiens growth differentiation factor 15 (GDF15), mRNA. |
| CDK5RAP2 | 10.65 | Homo sapiens CDK5 regulatory subunit associated protein 2 (CDK5RAP2), transcript variant 2, mRNA. |
| CDK5RAP2 | 10.54 | Homo sapiens CDK5 regulatory subunit associated protein 2 (CDK5RAP2), transcript variant 1, mRNA. |
| AFP | 9.83 | Homo sapiens alpha-fetoprotein (AFP), mRNA. |
| MIR199A2 | 9.30 | Homo sapiens microRNA 199a-2 (MIR199A2), microRNA. |
| PTGR1 | 9.25 | Homo sapiens prostaglandin reductase 1 (PTGR1), mRNA. |
| HSPA1A | 9.13 | Homo sapiens heat shock 70kDa protein 1A (HSPA1A), mRNA. |
| DDIT3 | 8.94 | Homo sapiens DNA-damage-inducible transcript 3 (DDIT3), mRNA. |
| GADD45A | 8.75 | Homo sapiens growth arrest and DNA-damage-inducible, alpha (GADD45A), mRNA. |
| SLCO2B1 | 8.61 | Homo sapiens solute carrier organic anion transporter family, member 2B1 (SLCO2B1), mRNA. |
| GADD45A | 8.38 | Homo sapiens growth arrest and DNA-damage-inducible, alpha (GADD45A), mRNA. |
| SLC3A2 | 8.36 | Homo sapiens solute carrier family 3 (activators of dibasic and neutral amino acid transport), member 2 (SLC3A2), transcript variant 6, mRNA. |
| SPP1 | 8.20 | Homo sapiens secreted phosphoprotein 1 (SPP1), transcript variant 2, mRNA. |
| TRIB3 | 8.20 | Homo sapiens tribbles homolog 3 (Drosophila) (TRIB3), mRNA. |
| SLC3A2 | 8.02 | Homo sapiens solute carrier family 3 (activators of dibasic and neutral amino acid transport), member 2 (SLC3A2), transcript variant 6, mRNA. |
| HSPA1B | 7.93 | Homo sapiens heat shock 70kDa protein 1B (HSPA1B), mRNA. |
| SPP1 | 7.91 | Homo sapiens secreted phosphoprotein 1 (SPP1), transcript variant 1, mRNA. |
| MMP10 | 7.76 | Homo sapiens matrix metallopeptidase 10 (stromelysin 2) (MMP10), mRNA. |
| MTHFD2L | 7.66 | Homo sapiens methylenetetrahydrofolate dehydrogenase (NADP+ dependent) 2-like (MTHFD2L), mRNA. |
| CCL26 | 7.64 | Homo sapiens chemokine (C-C motif) ligand 26 (CCL26), mRNA. |
| RDH10 | 7.64 | Homo sapiens retinol dehydrogenase 10 (all-trans) (RDH10), mRNA. |
| PTGR1 | 7.64 | Homo sapiens prostaglandin reductase 1 (PTGR1), mRNA. |
| GCLM | 7.54 | Homo sapiens glutamate-cysteine ligase, modifier subunit (GCLM), mRNA. |
| LOC100129186 | 7.35 | PREDICTED: Homo sapiens hypothetical protein LOC100129186 (LOC100129186), mRNA. |
| SLC3A2 | 7.34 | Homo sapiens solute carrier family 3 (activators of dibasic and neutral amino acid transport), member 2 (SLC3A2), transcript variant 1, mRNA. |
| SEL1L3 | 7.16 | Homo sapiens sel-1 suppressor of lin-12-like 3 (C. elegans) (SEL1L3), mRNA. |
| AKR1B10 | 6.22 | Homo sapiens aldo-keto reductase family 1, member B10 (aldose reductase) (AKR1B10), mRNA. |
| DHRS7 | 6.14 | Homo sapiens dehydrogenase/reductase (SDR family) member 7 (DHRS7), mRNA. |
| SLC12A8 | 6.13 | Homo sapiens solute carrier family 12 (potassium/chloride transporters), member 8 (SLC12A8), mRNA. |
| OSGIN1 | 6.12 | Homo sapiens oxidative stress induced growth inhibitor 1 (OSGIN1), transcript variant 2, mRNA. |
| AKR1C3 | 6.08 | Homo sapiens aldo-keto reductase family 1, member C3 (3-alpha hydroxysteroid dehydrogenase, type II) (AKR1C3), mRNA. |
| TSPAN13 | 5.95 | Homo sapiens tetraspanin 13 (TSPAN13), mRNA. |
| PIR | 5.90 | Homo sapiens pirin (iron-binding nuclear protein) (PIR), transcript variant 2, mRNA. |
| TNFSF18 | 5.86 | Homo sapiens tumor necrosis factor (ligand) superfamily, member 18 (TNFSF18), mRNA. |
| FLRT2 | 5.85 | Homo sapiens fibronectin leucine rich transmembrane protein 2 (FLRT2), mRNA. |
| DDX10 | 5.85 | Homo sapiens DEAD (Asp-Glu-Ala-Asp) box polypeptide 10 (DDX10), mRNA. |
| MAP2 | 5.56 | Homo sapiens microtubule-associated protein 2 (MAP2), transcript variant 1, mRNA. |
| TMEFF2 | 5.55 | Homo sapiens transmembrane protein with EGF-like and two follistatin-like domains 2 (TMEFF2), mRNA. |
| F3 | 5.44 | Homo sapiens coagulation factor III (thromboplastin, tissue factor) (F3), mRNA. |
| FLRT3 | 5.43 | Homo sapiens fibronectin leucine rich transmembrane protein 3 (FLRT3), transcript variant 2, mRNA. |
| GLA | 5.33 | Homo sapiens galactosidase, alpha (GLA), mRNA. |
| SRGN | -5.28 | Homo sapiens serglycin (SRGN), mRNA. |
| RFC4 | -5.32 | Homo sapiens replication factor C (activator 1) 4, 37kDa (RFC4), transcript variant 2, mRNA. |
| SOCS2 | -5.35 | Homo sapiens suppressor of cytokine signaling 2 (SOCS2), mRNA. |
| FAM20C | -5.43 | Homo sapiens family with sequence similarity 20, member C (FAM20C), mRNA. |
| STX11 | -5.49 | Homo sapiens syntaxin 11 (STX11), mRNA. |
| CCNA2 | -5.50 | Homo sapiens cyclin A2 (CCNA2), mRNA. |
| SERPINB7 | -5.52 | Homo sapiens serpin peptidase inhibitor, clade B (ovalbumin), member 7 (SERPINB7), transcript variant 1, mRNA. |
| COL4A2 | -5.54 | Homo sapiens collagen, type IV, alpha 2 (COL4A2), mRNA. |
| HMMR | -5.55 | Homo sapiens hyaluronan-mediated motility receptor (RHAMM) (HMMR), transcript variant 1, mRNA. |
| TMEM132A | -5.55 | Homo sapiens transmembrane protein 132A (TMEM132A), transcript variant 2, mRNA. |
| FAM43A | -5.56 | Homo sapiens family with sequence similarity 43, member A (FAM43A), mRNA. |
| MELK | -5.64 | Homo sapiens maternal embryonic leucine zipper kinase (MELK), mRNA. |
| CDCA7 | -5.69 | Homo sapiens cell division cycle associated 7 (CDCA7), transcript variant 1, mRNA. |
| SLC26A4 | -5.70 | Homo sapiens solute carrier family 26, member 4 (SLC26A4), mRNA. |
| MKX | -5.70 | Homo sapiens mohawk homeobox (MKX), mRNA. |
| NTN1 | -5.74 | Homo sapiens netrin 1 (NTN1), mRNA. |
| IL32 | -5.76 | Homo sapiens interleukin 32 (IL32), transcript variant 4, mRNA. |
| ZWINT | -5.81 | Homo sapiens ZW10 interactor (ZWINT), transcript variant 3, mRNA. |
| IGFBP7 | -5.86 | Homo sapiens insulin-like growth factor binding protein 7 (IGFBP7), mRNA. |
| SFRP2 | -5.86 | Homo sapiens secreted frizzled-related protein 2 (SFRP2), mRNA. |
| CCL3L1 | -5.87 | Homo sapiens chemokine (C-C motif) ligand 3-like 1 (CCL3L1), mRNA. |
| C15ORF48 | -5.92 | Homo sapiens chromosome 15 open reading frame 48 (C15orf48), transcript variant 2, mRNA. |
| PRC1 | -5.97 | Homo sapiens protein regulator of cytokinesis 1 (PRC1), transcript variant 2, mRNA. |
| HECW2 | -6.05 | Homo sapiens HECT, C2 and WW domain containing E3 ubiquitin protein ligase 2 (HECW2), mRNA. |
| SLC43A3 | -6.05 | Homo sapiens solute carrier family 43, member 3 (SLC43A3), mRNA. |
| MT1X | -6.08 | Homo sapiens metallothionein 1X (MT1X), mRNA. |
| NOD2 | -6.10 | Homo sapiens nucleotide-binding oligomerization domain containing 2 (NOD2), mRNA. |
| C10ORF58 | -6.13 | Homo sapiens chromosome 10 open reading frame 58 (C10orf58), transcript variant 1, mRNA. |
| NR4A2 | -6.16 | Homo sapiens nuclear receptor subfamily 4, group A, member 2 (NR4A2), transcript variant 1, mRNA. |
| FAM65C | -6.22 | Homo sapiens family with sequence similarity 65, member C (FAM65C), mRNA. |
| AK3L1 | -6.31 | Homo sapiens adenylate kinase 3-like 1 (AK3L1), nuclear gene encoding mitochondrial protein, transcript variant 6, mRNA. |
| CLIC6 | -6.35 | Homo sapiens chloride intracellular channel 6 (CLIC6), mRNA. |
| PLK4 | -6.36 | Homo sapiens polo-like kinase 4 (Drosophila) (PLK4), mRNA. |
| LIPG | -6.40 | Homo sapiens lipase, endothelial (LIPG), mRNA. |
| GRAMD1A | -6.43 | Homo sapiens GRAM domain containing 1A (GRAMD1A), mRNA. |
| GPM6B | -6.44 | Homo sapiens glycoprotein M6B (GPM6B), transcript variant 4, mRNA. |
| C16ORF75 | -6.50 | Homo sapiens chromosome 16 open reading frame 75 (C16orf75), mRNA. |
| AK3L1 | -6.54 | Homo sapiens adenylate kinase 3-like 1 (AK3L1), nuclear gene encoding mitochondrial protein, transcript variant 7, mRNA. |
| CA12 | -6.58 | Homo sapiens carbonic anhydrase XII (CA12), transcript variant 1, mRNA. |
| NR4A2 | -6.60 | Homo sapiens nuclear receptor subfamily 4, group A, member 2 (NR4A2), transcript variant 1, mRNA. |
| CENTA1 | -6.60 | Homo sapiens centaurin, alpha 1 (CENTA1), mRNA. |
| RAD51AP1 | -6.61 | Homo sapiens RAD51 associated protein 1 (RAD51AP1), mRNA. |
| NUSAP1 | -6.62 | Homo sapiens nucleolar and spindle associated protein 1 (NUSAP1), transcript variant 2, mRNA. |
| MCM2 | -6.63 | Homo sapiens minichromosome maintenance complex component 2 (MCM2), mRNA. |
| SERPINB7 | -6.65 | Homo sapiens serpin peptidase inhibitor, clade B (ovalbumin), member 7 (SERPINB7), transcript variant 1, mRNA. |
| TNFSF10 | -6.66 | Homo sapiens tumor necrosis factor (ligand) superfamily, member 10 (TNFSF10), mRNA. |
|  | -6.75 | Homo sapiens thymidine kinase 1, soluble (TK1), mRNA. |
| MMP12 | -6.75 | Homo sapiens matrix metallopeptidase 12 (macrophage elastase) (MMP12), mRNA. |
| C5ORF13 | -6.78 | Homo sapiens chromosome 5 open reading frame 13 (C5orf13), mRNA. |
| MYH11 | -6.84 | Homo sapiens myosin, heavy chain 11, smooth muscle (MYH11), transcript variant SM1A, mRNA. |
| COL4A1 | -6.98 | Homo sapiens collagen, type IV, alpha 1 (COL4A1), mRNA. |
| TYMS | -6.99 | Homo sapiens thymidylate synthetase (TYMS), mRNA. |
| TRIP13 | -6.99 | Homo sapiens thyroid hormone receptor interactor 13 (TRIP13), mRNA. |
| GBP5 | -7.13 | Homo sapiens guanylate binding protein 5 (GBP5), mRNA. |
| LRRN3 | -7.14 | Homo sapiens leucine rich repeat neuronal 3 (LRRN3), mRNA. |
| HLA-F | -7.16 | Homo sapiens major histocompatibility complex, class I, F (HLA-F), transcript variant 1, mRNA. |
| MMP12 | -7.17 | Homo sapiens matrix metallopeptidase 12 (macrophage elastase) (MMP12), mRNA. |
| CDC20 | -7.24 | Homo sapiens cell division cycle 20 homolog (S. cerevisiae) (CDC20), mRNA. |
| DNER | -7.27 | Homo sapiens delta/notch-like EGF repeat containing (DNER), mRNA. |
| SLC2A5 | -7.29 | Homo sapiens solute carrier family 2 (facilitated glucose/fructose transporter), member 5 (SLC2A5), mRNA. |
| PBK | -7.39 | Homo sapiens PDZ binding kinase (PBK), mRNA. |
| VCAM1 | -7.43 | Homo sapiens vascular cell adhesion molecule 1 (VCAM1), transcript variant 1, mRNA. |
| C15ORF48 | -7.44 | Homo sapiens chromosome 15 open reading frame 48 (C15orf48), transcript variant 2, mRNA. |
| MCM4 | -7.55 | Homo sapiens minichromosome maintenance complex component 4 (MCM4), transcript variant 1, mRNA. |
| CD38 | -7.59 | Homo sapiens CD38 molecule (CD38), mRNA. |
| HLA-DRA | -7.63 | Homo sapiens major histocompatibility complex, class II, DR alpha (HLA-DRA), mRNA. |
| MT1E | -7.68 | Homo sapiens metallothionein 1E (MT1E), mRNA. |
| SLC39A8 | -7.69 | Homo sapiens solute carrier family 39 (zinc transporter), member 8 (SLC39A8), transcript variant 1, mRNA. |
| UHRF1 | -7.71 | Homo sapiens ubiquitin-like with PHD and ring finger domains 1 (UHRF1), transcript variant 1, mRNA. |
| WNK4 | -7.74 | Homo sapiens WNK lysine deficient protein kinase 4 (WNK4), mRNA. |
| CX3CL1 | -7.79 | Homo sapiens chemokine (C-X3-C motif) ligand 1 (CX3CL1), mRNA. |
| RHOU | -7.90 | Homo sapiens ras homolog gene family, member U (RHOU), mRNA. |
| FRMD4A | -7.91 | Homo sapiens FERM domain containing 4A (FRMD4A), mRNA. |
| LOC730415 | -7.96 | PREDICTED: Homo sapiens hypothetical LOC730415, transcript variant 2 (LOC730415), mRNA. |
| C13ORF33 | -8.00 | Homo sapiens chromosome 13 open reading frame 33 (C13orf33), mRNA. |
| CA12 | -8.00 | Homo sapiens carbonic anhydrase XII (CA12), transcript variant 1, mRNA. |
| FAM20A | -8.02 | Homo sapiens family with sequence similarity 20, member A (FAM20A), mRNA. |
| ASCL2 | -8.03 | Homo sapiens achaete-scute complex homolog 2 (Drosophila) (ASCL2), mRNA. |
| MCM10 | -8.09 | Homo sapiens minichromosome maintenance complex component 10 (MCM10), transcript variant 2, mRNA. |
| UBE2C | -8.13 | Homo sapiens ubiquitin-conjugating enzyme E2C (UBE2C), transcript variant 3, mRNA. |
| SERPINE1 | -8.23 | Homo sapiens serpin peptidase inhibitor, clade E (nexin, plasminogen activator inhibitor type 1), member 1 (SERPINE1), mRNA. |
| LRRN3 | -8.27 | Homo sapiens leucine rich repeat neuronal 3 (LRRN3), transcript variant 1, mRNA. |
| UBE2C | -8.31 | Homo sapiens ubiquitin-conjugating enzyme E2C (UBE2C), transcript variant 6, mRNA. |
| THBS1 | -8.33 | Homo sapiens thrombospondin 1 (THBS1), mRNA. |
| EDNRA | -8.42 | Homo sapiens endothelin receptor type A (EDNRA), mRNA. |
| RRM2 | -8.51 | Homo sapiens ribonucleotide reductase M2 polypeptide (RRM2), mRNA. |
| CCL7 | -8.52 | Homo sapiens chemokine (C-C motif) ligand 7 (CCL7), mRNA. |
| HS.561679 | -8.52 | DA830074 PLACE1 Homo sapiens cDNA clone PLACE1004374 5, mRNA sequence |
| SEMA4D | -8.71 | Homo sapiens sema domain, immunoglobulin domain (Ig), transmembrane domain (TM) and short cytoplasmic domain, (semaphorin) 4D (SEMA4D), mRNA. |
| SEPT4 | -8.82 | Homo sapiens septin 4 (SEPT4), transcript variant 2, mRNA. |
| G0S2 | -9.05 | Homo sapiens G0/G1switch 2 (G0S2), mRNA. |
| HSD11B1 | -9.07 | Homo sapiens hydroxysteroid (11-beta) dehydrogenase 1 (HSD11B1), transcript variant 2, mRNA. |
| METTL7A | -9.11 | Homo sapiens methyltransferase like 7A (METTL7A), mRNA. |
| HSD11B1 | -9.13 | Homo sapiens hydroxysteroid (11-beta) dehydrogenase 1 (HSD11B1), transcript variant 1, mRNA. |
| TGM2 | -9.34 | Homo sapiens transglutaminase 2 (C polypeptide, protein-glutamine-gamma-glutamyltransferase) (TGM2), transcript variant 1, mRNA. |
| HSD11B1 | -9.41 | Homo sapiens hydroxysteroid (11-beta) dehydrogenase 1 (HSD11B1), transcript variant 2, mRNA. |
| GAS1 | -9.48 | Homo sapiens growth arrest-specific 1 (GAS1), mRNA. |
| H2AFY2 | -9.58 | Homo sapiens H2A histone family, member Y2 (H2AFY2), mRNA. |
| MAD2L1 | -9.66 | Homo sapiens MAD2 mitotic arrest deficient-like 1 (yeast) (MAD2L1), mRNA. |
| SLC39A8 | -9.78 | Homo sapiens solute carrier family 39 (zinc transporter), member 8 (SLC39A8), transcript variant 1, mRNA. |
| GINS2 | -9.79 | Homo sapiens GINS complex subunit 2 (Psf2 homolog) (GINS2), mRNA. |
| CDCA5 | -10.00 | Homo sapiens cell division cycle associated 5 (CDCA5), mRNA. |
| AKAP12 | -10.03 | Homo sapiens A kinase (PRKA) anchor protein (gravin) 12 (AKAP12), transcript variant 1, mRNA. |
| MCM6 | -10.15 | Homo sapiens minichromosome maintenance complex component 6 (MCM6), mRNA. |
| CDC2 | -10.22 | Homo sapiens cell division cycle 2, G1 to S and G2 to M (CDC2), transcript variant 1, mRNA. |
| CFB | -10.39 | Homo sapiens complement factor B (CFB), mRNA. |
| TOP2A | -10.55 | Homo sapiens topoisomerase (DNA) II alpha 170kDa (TOP2A), mRNA. |
| PTGES | -10.60 | Homo sapiens prostaglandin E synthase (PTGES), mRNA. |
| AKAP12 | -10.72 | Homo sapiens A kinase (PRKA) anchor protein (gravin) 12 (AKAP12), transcript variant 2, mRNA. |
| GNA15 | -10.82 | Homo sapiens guanine nucleotide binding protein (G protein), alpha 15 (Gq class) (GNA15), mRNA. |
| RARRES1 | -10.84 | Homo sapiens retinoic acid receptor responder (tazarotene induced) 1 (RARRES1), transcript variant 1, mRNA. |
| RARRES1 | -10.88 | Homo sapiens retinoic acid receptor responder (tazarotene induced) 1 (RARRES1), transcript variant 2, mRNA. |
| HS.10862 | -10.90 | Homo sapiens cDNA: FLJ23313 fis, clone HEP11919 |
| CCL3L1 | -10.93 | Homo sapiens chemokine (C-C motif) ligand 3-like 1 (CCL3L1), mRNA. |
| SHRM | -11.06 | Homo sapiens shroom (SHRM), mRNA. |
| LPPR4 | -11.21 | Homo sapiens plasticity related gene 1 (LPPR4), mRNA. |
| GAS1 | -11.24 | Homo sapiens growth arrest-specific 1 (GAS1), mRNA. |
| CXCL6 | -11.51 | Homo sapiens chemokine (C-X-C motif) ligand 6 (granulocyte chemotactic protein 2) (CXCL6), mRNA. |
| CXCL6 | -11.59 | Homo sapiens chemokine (C-X-C motif) ligand 6 (granulocyte chemotactic protein 2) (CXCL6), mRNA. |
| CXCR7 | -11.73 | Homo sapiens chemokine (C-X-C motif) receptor 7 (CXCR7), mRNA. |
| NCAPG | -11.92 | Homo sapiens non-SMC condensin I complex, subunit G (NCAPG), mRNA. |
| C20ORF127 | -12.22 | Homo sapiens chromosome 20 open reading frame 127 (C20orf127), mRNA. |
| GPM6B | -13.34 | Homo sapiens glycoprotein M6B (GPM6B), transcript variant 1, mRNA. |
| PSTPIP2 | -13.50 | Homo sapiens proline-serine-threonine phosphatase interacting protein 2 (PSTPIP2), mRNA. |
| THBS2 | -13.96 | Homo sapiens thrombospondin 2 (THBS2), mRNA. |
| MT1G | -14.12 | Homo sapiens metallothionein 1G (MT1G), mRNA. |
| CCL3 | -14.36 | Homo sapiens chemokine (C-C motif) ligand 3 (CCL3), mRNA. |
| CDC45L | -14.85 | Homo sapiens CDC45 cell division cycle 45-like (S. cerevisiae) (CDC45L), mRNA. |
| CXCR7 | -14.92 | Homo sapiens chemokine (C-X-C motif) receptor 7 (CXCR7), transcript variant 1, mRNA. |
| MT1JP | -15.71 | Homo sapiens metallothionein 1J (pseudogene) (MT1JP), mRNA. |
| PI3 | -16.06 | Homo sapiens peptidase inhibitor 3, skin-derived (SKALP) (PI3), mRNA. |
| HAS3 | -17.26 | Homo sapiens hyaluronan synthase 3 (HAS3), transcript variant 1, mRNA. |
| KIAA0101 | -17.30 | Homo sapiens KIAA0101 (KIAA0101), transcript variant 1, mRNA. |
| GPM6B | -18.45 | Homo sapiens glycoprotein M6B (GPM6B), transcript variant 1, mRNA. |
| CCL3L3 | -18.86 | Homo sapiens chemokine (C-C motif) ligand 3-like 3 (CCL3L3), mRNA. |
| VCAM1 | -20.34 | Homo sapiens vascular cell adhesion molecule 1 (VCAM1), transcript variant 1, mRNA. |
| CD74 | -21.65 | Homo sapiens CD74 molecule, major histocompatibility complex, class II invariant chain (CD74), transcript variant 2, mRNA. |
| HIST1H4C | -21.76 | Homo sapiens histone cluster 1, H4c (HIST1H4C), mRNA. |
| CXCL9 | -22.73 | Homo sapiens chemokine (C-X-C motif) ligand 9 (CXCL9), mRNA. |
| CD74 | -23.45 | Homo sapiens CD74 molecule, major histocompatibility complex, class II invariant chain (CD74), transcript variant 1, mRNA. |
| CCL5 | -32.20 | Homo sapiens chemokine (C-C motif) ligand 5 (CCL5), mRNA. |
| CCL5 | -33.03 | Homo sapiens chemokine (C-C motif) ligand 5 (CCL5), mRNA. |
| MT1H | -36.15 | Homo sapiens metallothionein 1H (MT1H), mRNA. |
| XIRP1 | -37.11 | Homo sapiens xin actin-binding repeat containing 1 (XIRP1), mRNA. |
| HLA-DRA | -39.19 | Homo sapiens major histocompatibility complex, class II, DR alpha (HLA-DRA), mRNA. |
| MT1F | -43.80 | Homo sapiens metallothionein 1F (MT1F), mRNA. |
| UBD | -44.02 | Homo sapiens ubiquitin D (UBD), mRNA. |
| MT1M | -86.45 | Homo sapiens metallothionein 1M (MT1M), mRNA. |

Table S4. Top 20 genes regulated by CBEO in the canonical Mitotic Roles of Polo-Like Kinase pathway. Fold change over vehicle was shown in log_2_ ratio form.


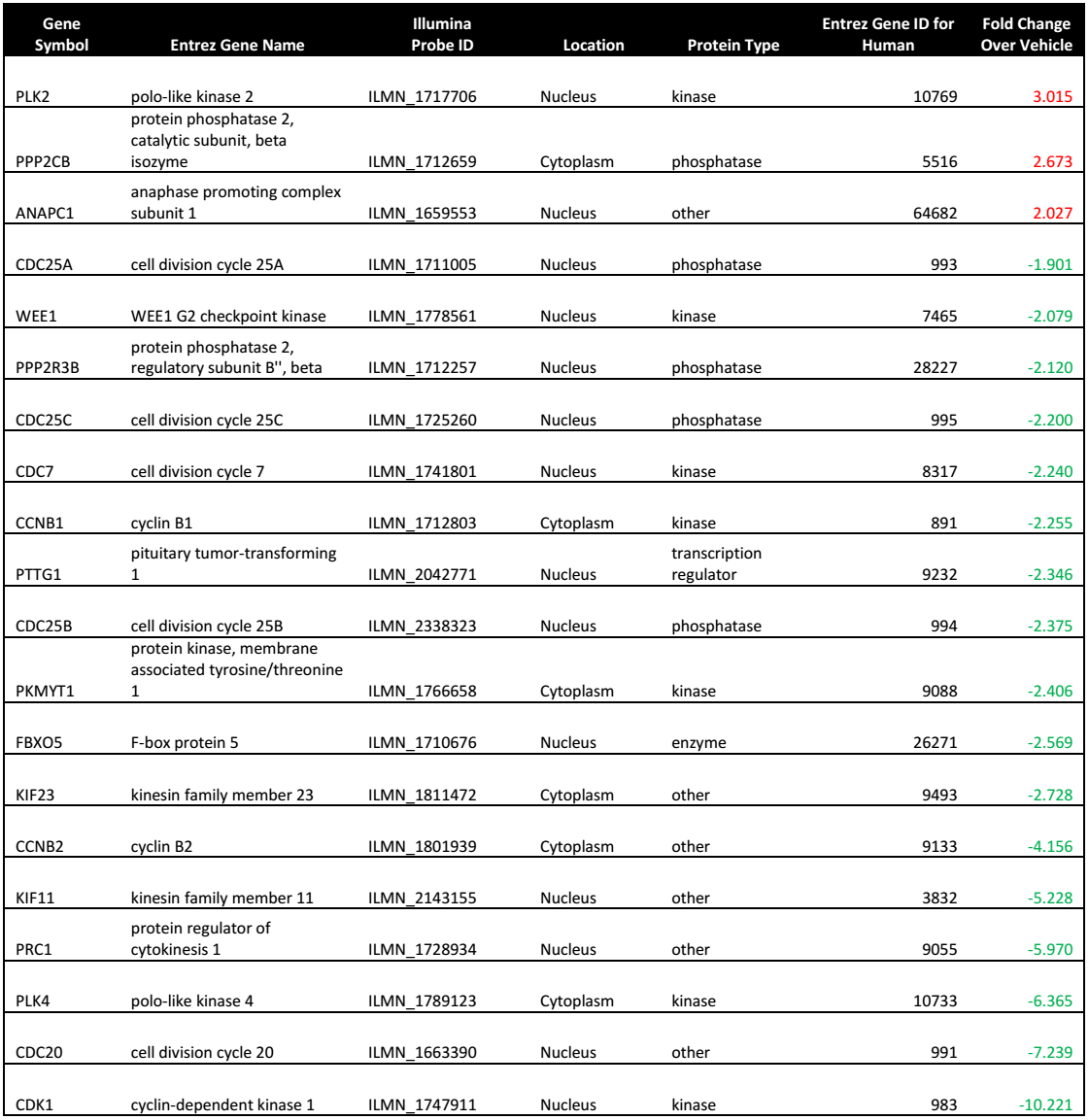


Table S5. Top 15 genes regulated by CBEO in the canonical Mismatch Repair in Eukaryotes pathway. Fold change over vehicle was shown in log_2_ ratio form.


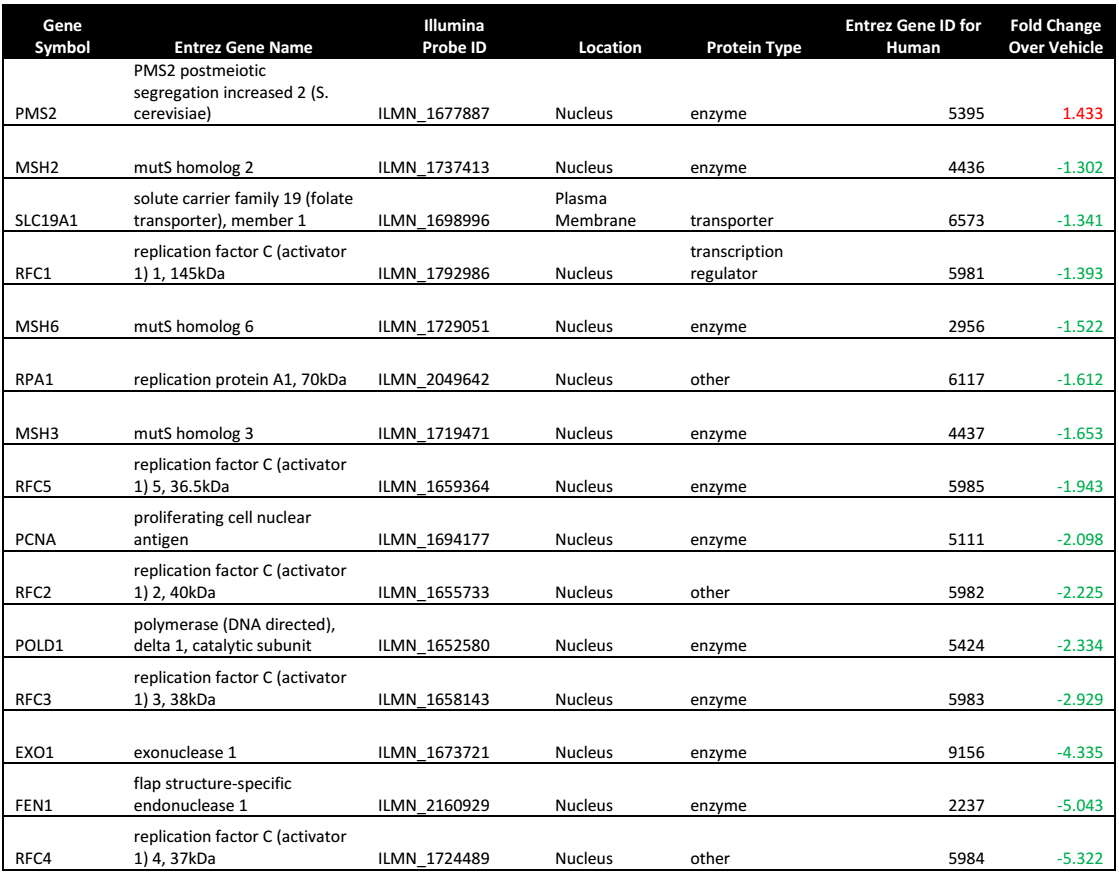


Table S6. Top 20 genes regulated by CBEO in the canonical Protein Ubiquitination pathway. Fold change over vehicle was shown in log_2_ ratio form.


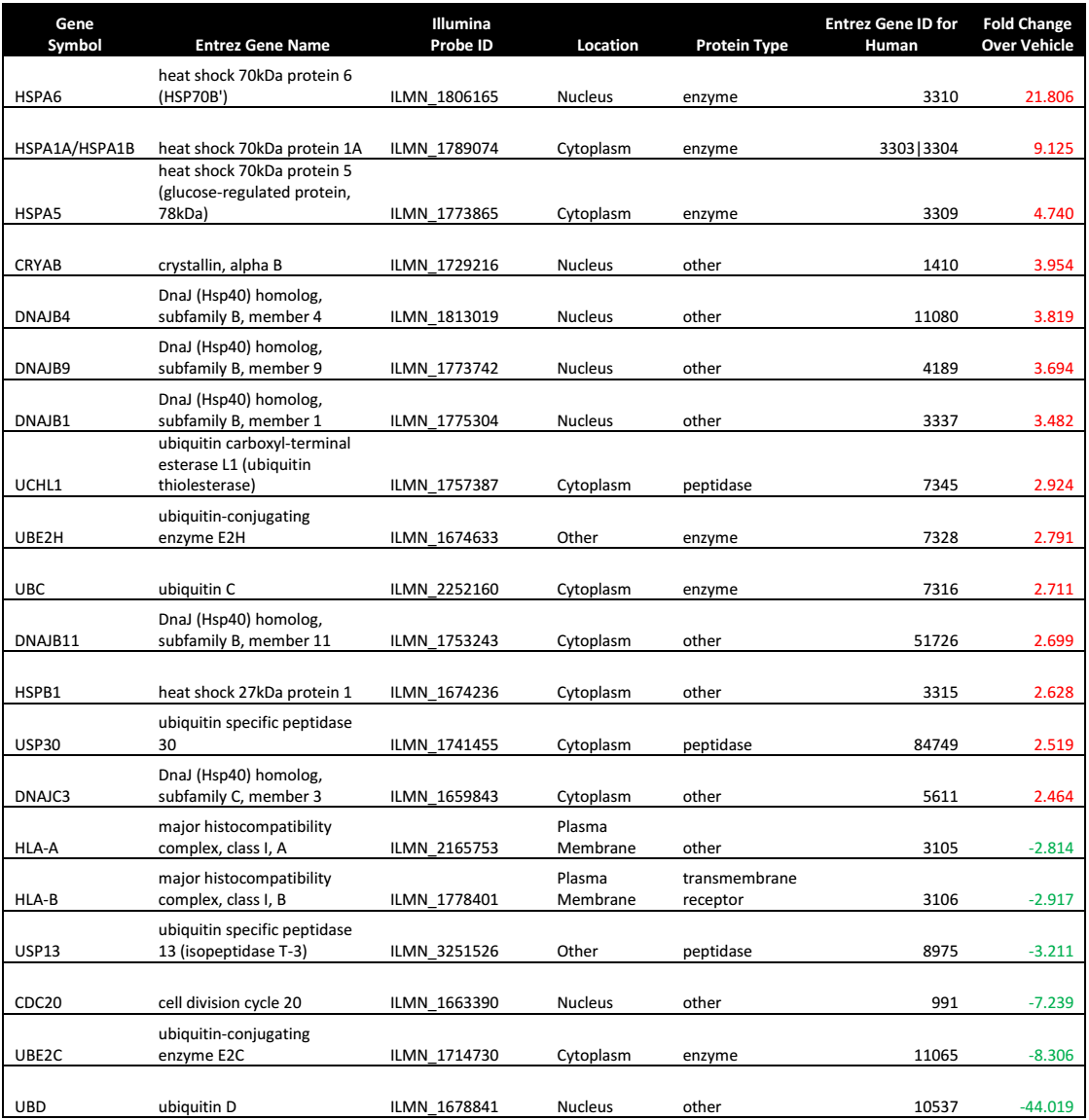


Table S7. Top 20 genes regulated by CBEO in the canonical NRF2-mediated Oxidative Stress Response pathway. Fold change over vehicle was shown in log_2_ ratio form.
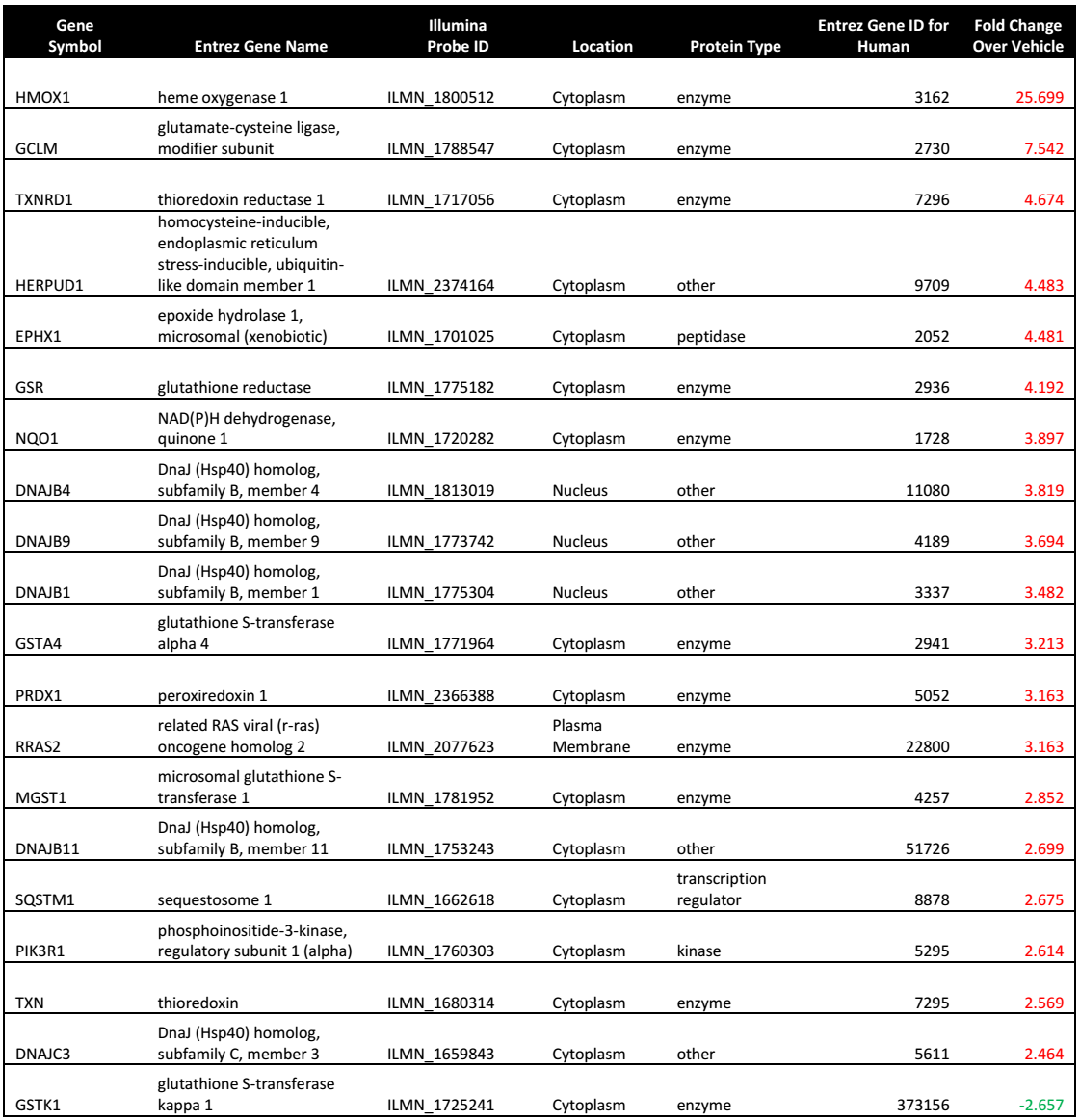

Supplement: Supplementary file 1 — Fig. S1. The chromatogram of cinnamon bark essential oil (CBEO) analyzed by gas chromatography–mass spectrometry (GC–MS). Table S1. Chemical composition of cinnamon bark essential oil (CBEO) analyzed by gas chromatography–mass spectrometry (GC–MS). Table S2. Glossary of biomarkers of system HDF3CGF used in the study. Table S3. The 200 genes most‐impacted by cinnamon bark essential oil (CBEO). Table S4. Top 20 genes regulated by CBEO in the canonical Mitotic Roles of Polo‐Like Kinase pathway. Fold change over vehicle was shown in log2 ratio form. Table S5. Top 15 genes regulated by CBEO in the canonical Mismatch Repair in Eukaryotes pathway. Fold change over vehicle was shown in log2 ratio form. Table S6. Top 20 genes regulated by CBEO in the canonical Protein Ubiquitination pathway. Fold change over vehicle was shown in log2 ratio form. Table S7. Top 20 genes regulated by CBEO in the canonical NRF2‐mediated Oxidative Stress Response pathway. Fold change over vehicle was shown in log2 ratio form. [file PTR-31-1034-s001.docx]
